# Structural and Functional Coupling of Calcium-Activated BK Channels and Calcium-Permeable Channels Within Nanodomain Signaling Complexes

**DOI:** 10.3389/fphys.2021.796540

**Published:** 2022-01-14

**Authors:** Kunal R. Shah, Xin Guan, Jiusheng Yan

**Affiliations:** ^1^Department of Anesthesiology & Perioperative Medicine, The University of Texas MD Anderson Cancer Center, Houston, TX, United States; ^2^Neuroscience Program, Graduate School of Biomedical Sciences, UT Health, The University of Texas MD Anderson Cancer Center, Houston, TX, United States; ^3^Biochemistry and Cell Biology Program, Graduate School of Biomedical Sciences, UT Health, The University of Texas MD Anderson Cancer Center, Houston, TX, United States

**Keywords:** BK channels, calcium-activated channels, calcium channels, NMDA receptors, nanodomain, coupling, calcium signaling

## Abstract

Biochemical and functional studies of ion channels have shown that many of these integral membrane proteins form macromolecular signaling complexes by physically associating with many other proteins. These macromolecular signaling complexes ensure specificity and proper rates of signal transduction. The large-conductance, Ca^2+^-activated K^+^ (BK) channel is dually activated by membrane depolarization and increases in intracellular free Ca^2+^ ([Ca^2+^]_i_). The activation of BK channels results in a large K^+^ efflux and, consequently, rapid membrane repolarization and closing of the voltage-dependent Ca^2+^-permeable channels to limit further increases in [Ca^2+^]_i_. Therefore, BK channel-mediated K^+^ signaling is a negative feedback regulator of both membrane potential and [Ca^2+^]_i_ and plays important roles in many physiological processes and diseases. However, the BK channel formed by the pore-forming and voltage- and Ca^2+^-sensing α subunit alone requires high [Ca^2+^]_i_ levels for channel activation under physiological voltage conditions. Thus, most native BK channels are believed to co-localize with Ca^2+^-permeable channels within nanodomains (a few tens of nanometers in distance) to detect high levels of [Ca^2+^]_i_ around the open pores of Ca^2+^-permeable channels. Over the last two decades, advancement in research on the BK channel’s coupling with Ca^2+^-permeable channels including recent reports involving NMDA receptors demonstrate exemplary models of nanodomain structural and functional coupling among ion channels for efficient signal transduction and negative feedback regulation. We hereby review our current understanding regarding the structural and functional coupling of BK channels with different Ca^2+^-permeable channels.

## Introduction

### Ca^2+^ and K^+^ Signaling *via* Ion Channels

Cells need to sense and respond to changes in the extracellular environment and communicate with adjoining and distant cells. Cells use different signaling molecules to carry out these tasks. Ca^2+^ and K^+^ cations are two signaling molecules that are abundant and used for cell signaling in all living systems. Ca^2+^ is a prominent second messenger molecule that is involved in nearly all biochemical, cellular, and physiological processes. It is essential for proper cardiac function, the structural integrity of bone, and muscular contraction, and it acts as a substrate for enzymatic signal in biochemical pathways. Cells invest vast amounts of energy into the regulation of intracellular concentrations of free Ca^2+^. The speed and effectiveness of Ca^2+^ signaling builds upon the greater than 10,000-fold gradient between the Ca^2+^ concentrations inside (∼100 nM) and outside (∼2 mM) the cells. Rapid global (10-fold) and local (100-fold) increases in intracellular free Ca^2+^ ([Ca^2+^]_i_) concentrations can be achieved by Ca^2+^ influx from extracellular mediums or Ca^2+^ release from intracellular Ca^2+^ stores *via* the activation of Ca^2+^-permeable channels on the plasma membrane and intracellular organelle membranes, respectively.

Ca^2+^-permeable channels passively diffuse Ca^2+^ ions down their electrochemical gradient. There is a wide variety of Ca^2+^-permeable channels that differ in Ca^2+^ selectivity and activation mechanisms. Ca^2+^-selective channels include voltage-gated Ca^2+^ (Ca_V_) channels, including Ca_V_1.1-4, Ca_V_2.1-3, and Ca_V_3.1-3; ligand-gated Ca^2+^ channels, including the store-operated Ca^2+^ channels (SOCCs or ORAI1-3) on the plasma membrane; and inositol 1,4,5-trisphosphate (IP3) and ryanodine receptors (IP3Rs and RyRs) on the endoplasmic reticulum (ER) membrane. Non-selective cation channels include mechanosensitive piezo channels, transient receptor potential (TRP) channels, cyclic nucleotide–gated ion channels (CNGs), acid-sensing ion channels (ASICs), and ionotropic receptors, including *N*-methyl-D-aspartate (NMDA) receptors, serotonin 5-hydroxytryptamine (HT3) receptors, and adenosine triphosphate (ATP)–activated P2X receptors.

K^+^ is the most abundant cation in the intracellular fluid. The concentration of K^+^ ions is usually about 25-fold higher on the cytoplasmic than on the extracellular side of the plasma membrane. K^+^ channels selectively pass K^+^ ions across membrane according to electrochemical driving forces. Channel-mediating K^+^ signaling dynamically controls K^+^ distribution across the cell membrane and is critical for normal cell function. The K^+^ channel current dominates the ionic flow in a cell’s resting state and thus is critical for setting a cell’s resting membrane potential. K^+^ channels are also involved in fluid secretion and cell volume regulation. In excitable cells, the K^+^ channel’s activities affect the action potential firing threshold, which is determined by the balance between inward Na^+^ and outward K^+^ currents and also underlies the repolarization, hyperpolarization, and after-hyperpolarization phases of the action potential. K^+^ channels play a crucial role in all aspects of life by regulating the excitability of neurons and the heart, contracting muscles, secreting hormones, water homeostasis, and activating immune cells.

K^+^ channels are the most diverse and abundantly expressed ion channels in living organisms. These channels are expressed in most excitable and non-excitable cells and perform numerous important functions, as is evident from the large number of genes (∼80 in mammals) encoding for the K^+^ channels’ pore-forming subunits. K^+^ channels can be classified into four main groups: Ca^2+–^activated K^+^ (K_Ca_) channels, tandem pore domain K^+^ (K_2_P) channels, voltage-gated K^+^ (Kv) channels, and inwardly rectifying K^+^ (Kir) channels. Each type of K^+^ channel possesses unique electrophysiological and pharmacological properties. K_Ca_ channels activate in response to increases in [Ca^2+^]_i_ and thus cause changes in cell membrane potential toward the negative voltage direction *via* K^+^ efflux. This common functionality enables K_Ca_ channels to play an important role in bridging cell excitability and the intracellular calcium concentration. K_Ca_ channels are a diversified group of channels with various biophysical and pharmacological properties. K_Ca_ channels are divided into 3 classes based on their single channel conductance: big conductance (BK, 200–300 pS), intermediate conductance (IK, 32–39 pS), and small conductance (SK, 4–14 pS).

### BK Channels: General Properties and Function

**FIGURE 1:**
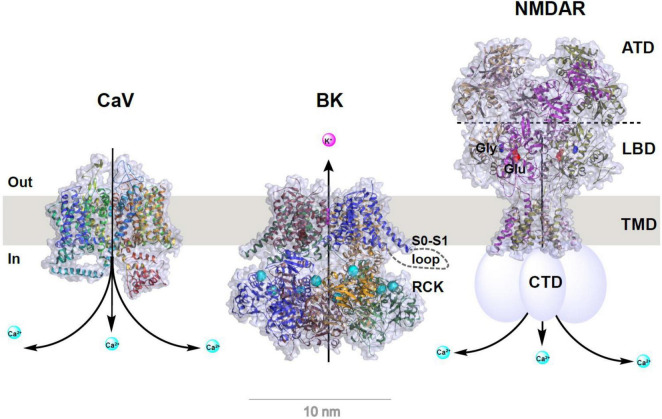
Structural models of Ca_V_ and BK channels and NMDA receptor. Structures were redrawn from published structures of rabbit Ca_V_1.1 (PDB ID: 5GJV) (Wu et al., 2016), human BK (PDB ID: 6V22) (Tao and MacKinnon, 2019), and rat GluN1a/GluN2B NMDA receptor (PDB ID: 4PE5) (Karakas and Furukawa, 2014). For clarity, auxiliary subunits of Ca_V_ and BK channels are omitted and the bound Ca^2+^ ions in BK channel’s RCK domains are shown as enlarged cyan balls. Depiction in cartoon was added manually for the S0–S1 loop (only on one subunit) of BK channel and C-terminal domain (CTD) of NMDA receptor whose structures were unresolved. For NMDA receptor, the amino terminal domain (ATD), ligand binding domain (LBD), transmembrane domain (TMD), the bound glutamate (Glu) and glycine (Gly) are indicated on or near the corresponding structural parts.

The BK channel (also known as K_Ca_1.1, MaxiK, and slo1) is a homotetrameric channel consisting of four identical subunits of the pore-forming, Ca^2+^- and voltage-sensing α-subunit (BKα, encoded by a single gene *KCNMA1*) either alone or in association with regulatory β or γ subunits. The BKα channel (∼130 kDa) contains 7 transmembrane (TM) segments (S0–S6), a short extracellular N-terminus, and a large cytosolic C-terminus composed of two regulating conductance of K^+^ (RCK) domains for Ca^2+^ sensing (Tao and MacKinnon, 2019) (Figure 1). The four auxiliary β subunits (β1–β4) and the four γ subunits (γ1–γ4) are double- and single-spanning membrane proteins, respectively, with tissue-specific expression patterns. For example, β1 is mainly in smooth muscles, γ1 is in secretory, non-excitable cells, and γ3 and β4 are in the brain (Solaro and Lingle, 1992; Behrens et al., 2000; Brenner et al., 2000; Cox and Aldrich, 2000; Yan and Aldrich, 2010, 2012; Zhang and Yan, 2014).

BK channels have an exceptionally large single-channel conductance, which is 10–20 times larger than that of most other K^+^ channels. BK channel activation is regulated by membrane voltage and a wide range of [Ca^2+^]_i_ (from sub-micromole to hundreds of micromoles) *via* Ca^2+^ bindings at the RCK domains. When activated by Ca^2+^ channel–mediated [Ca^2+^]_i_ elevation, BK channels generate large K^+^ efflux and, consequently, rapid membrane repolarization that can limit further Ca^2+^ flux through membrane repolarization–induced deactivation of Ca^2+^ channels. *Via* this negative feedback mechanism, BK channel–mediated K^+^ signaling plays a powerful, integrative role in regulating cellular excitability and calcium signaling in electrically excitable cells (Ghatta et al., 2006; Salkoff et al., 2006).

The BK channels are critically involved in various cellular and physiological processes. In central neurons, BK channels mediate the repolarization and fast after hyperpolarization (fAHP) of action potentials (Shao et al., 1999; Womack and Khodakhah, 2002), shape dendritic Ca^2+^ spikes (Golding et al., 1999), and regulate neurotransmitter release at presynaptic terminals (Hu et al., 2001; Raffaelli et al., 2004; Xu and Slaughter, 2005; Samengo et al., 2014). In addition, they are involved in motor coordination (Sausbier et al., 2004), learning and memory (Matthews and Disterhoft, 2009; Ye et al., 2010; Typlt et al., 2013; Springer et al., 2014), the brain’s intrinsic rhythmicity of the circadian clock (Meredith et al., 2006; Pitts et al., 2006; Montgomery et al., 2013; Farajnia et al., 2015) and respiration (Onimaru et al., 2003; Zhao et al., 2006; Zavala-Tecuapetla et al., 2008), frequency tuning of cochlear hair cells (Fettiplace and Fuchs, 1999), pain modulation (Chen et al., 2009; Cao et al., 2012; Zhang et al., 2012; Waxman and Zamponi, 2014), and neuroprotection under pathological conditions (Runden-Pran et al., 2002; Shen et al., 2007; Zhang et al., 2009; Mancini et al., 2014). Defects in or dysregulation of human neuronal BK channels can cause epilepsy and paroxysmal dyskinesia (Brenner et al., 2005; Du et al., 2005) and are implicated in intellectual disability (Higgins et al., 2008; Deng et al., 2013), autism (Laumonnier et al., 2006), and schizophrenia (Zhang et al., 2006).

### The Necessity and Properties of Nanodomain Coupling of BK Channels With Ca^2+^-Permeable Channels

**FIGURE 2:**
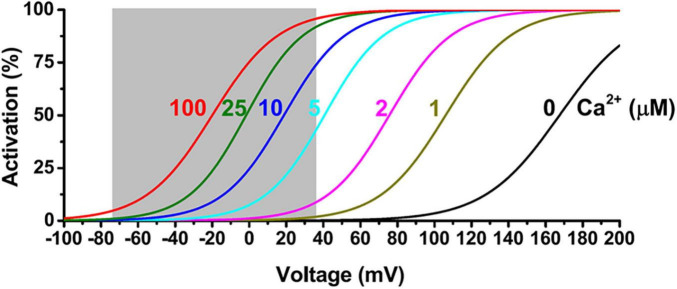
Voltage and Ca^2+^ dependence of BKα channel activation. The shaded area indicates a physiological range of membrane voltages.

In spite of multimode activation by cell membrane depolarization, a rise in [Ca^2+^]_i_, or both synergistically, BK channels are generally considered high-threshold channels. For the BK channels formed by the α subunit alone, a very high voltage of more than 100 mV, which is out of the physiological range, is needed for measurable BK channel activation under the cell’s resting Ca^2+^ conditions (≤∼0.1 μM) (Figure 2). Under resting membrane voltage conditions (e.g., −60 to −80 mV), an extremely high (≥100 μM) concentration of [Ca^2+^]_i_ is needed to produce channel activation (Figure 2). Under membrane depolarization conditions in excitable neuronal (Vm ≤ +40 mV) or smooth muscle cells (Vm ≤ +20 mV) during action potential, the required [Ca^2+^]_i_ concentration for significant BK channel activation is also approximately a few μM, which is generally higher than the cellular global [Ca^2+^]_i_ concentration in resting (≤∼0.1 μM) and excited (≤∼1 μM) states (Figure 2). In contrast, the other two types of K_Ca_ channels, IK and SK, have a much higher affinity for Ca^2+^ (EC_50_ ≈ 0.3–0.5 μM) owing to their constitutively bound Ca^2+^-binding messenger protein, calmodulin (CaM). Therefore, BK channels had been considered to function mainly as a brake operating only under extreme conditions, e.g., a pathological [Ca^2+^]_i_ overload repolarizing the membrane to limit further Ca^2+^ influx. Research progress in the last 2 decades has revealed that cells employ at least two effective biochemical strategies to allow BK channels to be activated under normal cellular or physiological conditions.

The first strategy is to modulate the voltage dependence of the channel activation *via* auxiliary proteins. The γ1 (LRRC26) subunit in particular gives BK channels an unusual capability to be constitutively active at physiological voltages and [Ca^2+^]_i_ levels in non-excitable cells by causing a large negative shift (∼−140 mV) in the voltage dependence of the channel activation (Yan and Aldrich, 2010, 2012). The γ1 subunit is highly expressed in secretory epithelia cells in different organs and plays an important role in the resting K^+^ efflux and fluid secretion in these cells (Yang et al., 2017; Gonzalez-Perez et al., 2021). The γ2 subunit also results in a great shift (∼−100 mV) in BK channel voltage gating (Yan and Aldrich, 2012). The γ2 subunit is highly expressed in the testis and is a potent regulator of BK channel function in the cochlea’s inner hair cells (Lingle, 2019). The β subunits overall do not strongly affect the thresholds of BK channel activation, despite their complex effects on BK channel voltage and Ca^2+^ gating and current kinetics. A detailed review of the BK channel β and γ subunits is beyond the scope of this work and can be found in previous articles (Zhang and Yan, 2014; Li and Yan, 2016; Latorre et al., 2017; Gonzalez-Perez and Lingle, 2019). The second strategy that cells use to activate BK channels is to position BK channels in proximity to some Ca^2+^-permeable channels to gain immediate access to the local high concentrations of [Ca^2+^]_i_ during extracellular Ca^2+^ influx or Ca^2+^ release from intracellular Ca^2+^ store organelles.

**FIGURE 3:**
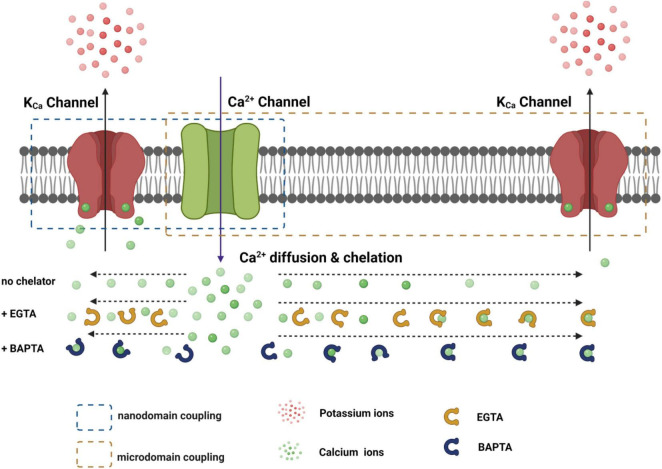
Functional coupling of the K_Ca_ channel with the Ca^2+^ channel within nanodomain and microdomain. The distance-dependent effects of EGTA and BATPA on the availability of free Ca^2+^ ions during their diffusion from the Ca^2+^ channel pore are roughly depicted.

**FIGURE 4:**
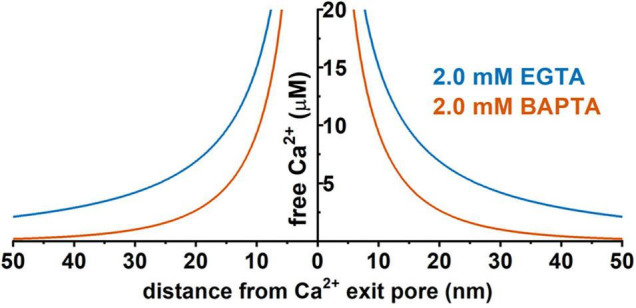
Predicted distance dependence of the local free Ca^2+^ concentration originating from a hypothetic Ca^2+^ channel pore in the presence of 2 mM EGTA or 2 mM BAPTA. Prediction was performed using the CalC software (version 6.8.0) (Matveev et al., 2004) at 0.1 pA for the single-channel Ca^2+^ current. Chelator and Ca^2+^ parameters were taken from previous reports (Naraghi and Neher, 1997; Fakler and Adelman, 2008; Eggermann et al., 2011).

Given the presence of endogenous Ca^2+^ buffers and the quick diffusion of Ca^2+^ ions, the distance to the Ca^2+^-originating site determines the local [Ca^2+^]_i_ concentration. Thus, rapid and precise spatiotemporal control of the [Ca^2+^]_i_ concentration is found in local Ca^2+^-signaling domains, in which Ca^2+^ channels and Ca^2+^-effector sensors are within proximity, i.e., within the nanodomain or microdomain (Augustine et al., 2003; Eggermann et al., 2011) (Figure 3). In Ca^2+^ signaling, a nanodomain is a conceptual region of highly localized Ca^2+^ signals extending over a few tens of nm from the cytoplasmic mouths of the Ca^2+^ channels (Eggermann et al., 2011; Tay et al., 2012). Coupling between Ca^2+^ sensors and Ca^2+^ channels within nanodomains can achieve high efficacy, speed, and energy efficiency for Ca^2+^ signaling (Eggermann et al., 2011). Experimentally, the colocalization of Ca^2+^ channels and Ca^2+^-effector sensors can be functionally probed with Ca^2+^ chelators, ethylene glycol tetraacetic acid (EGTA), and 1,2-bis(o-aminophenoxy)ethane-*N*,*N*,*N*′,*N*′-tetraacetic acid (BAPTA) (Figure 4). Both EGTA and BAPTA compete with Ca^2+^-sensing proteins for [Ca^2+^]_i_ and have similar steady-state binding affinities for Ca^2+^. However, they differ greatly in their rates of Ca^2+^ binding (Naraghi and Neher, 1997). BAPTA has a binding rate constant 150 times higher than that of EGTA. At millimolar levels, BAPTA can efficiently prevent the spreading of free Ca^2+^ from the entry site. The slower chelator, EGTA, is relatively ineffective in sequestering Ca^2+^ within a short distance of the Ca^2+^ exit site but can intercept Ca^2+^ during Ca^2+^ diffusion over a longer distance. For convenience, the local Ca^2+^ signaling domain is classified as a Ca^2+^ nanodomain or microdomain depending on the sensitivity of the Ca^2+^-sensing effector to EGTA and BAPTA. The Ca^2+^ nanodomain signaling process is effectively disrupted by millimolar levels (e.g., 2–10 mM) of BAPTA but not of EGTA, whereas in Ca^2+^ microdomains (e.g., from a few tens to a few hundred nm), EGTA also dissipates the signaling process effectively. Biochemically, the nanodomain or microdomain coupling of Ca^2+^-sensing proteins to Ca^2+^ channels can be achieved by direct protein-protein physical interactions for nanodomain colocalization; indirect interactions mediated by other proteins, e.g., scaffold proteins (Sclip et al., 2018), for nano- or microdomain colocalization; and special membrane domains, e.g., lipid rafts (Ma et al., 2015), that concentrate and restrict their distribution for nano- or microdomain colocalization.

The distance to the Ca^2+^ source is a key factor determining BK channel activation and function. Given the presence of endogenous cytosolic Ca^2+^ chelating buffer that can be comparable to EGTA in capacity (Fakler and Adelman, 2008; Schwaller, 2010), the microdomain coupling is expected to be largely ineffective in BK channel activation. For example, in the presence of 2 mM EGTA, a BK channel must be within 50 nm of a Ca^2+^ source to sense an effective local [Ca^2+^]_i_ concentration of at least 2 μM (Figure 4) in order to achieve significant activation, e.g., an open probability (*P*_*o*_) of 0.1 or greater, even at a maximally depolarized membrane voltage of +40 mV (Figure 2). Thus, under physiological conditions of limited membrane depolarization, native BK channels, particularly those in excitable cells that lack γ1 and γ2 expression, must colocalize with Ca^2+^ sources within nanodomains to be physiologically active and functional. Compared to other K_Ca_ channels, the requirement of nanodomain colocalization with Ca^2+^ source is specific to BK channels because the IK and SK channels’ exquisitely high affinity for Ca^2+^ theoretically relieves them from the requirement of close nanodomain interaction with Ca^2+^ sources for functional coupling, even in the presence of EGTA (Fakler and Adelman, 2008).

From the above discussion, BK channels coupled with Ca^2+^ channels within the nanodomain are expected to demonstrate: (1) the Ca^2+^ channel–mediated BK channel currents that are largely resistant to Ca^2+^ chelating by millimolar levels of EGTA but could be fully or partially sensitive to millimolar levels of BAPTA; (2) physical formation of protein complex by the 2 channels that can be copurified or coimmunoprecipitated in the forms of channel-channel complexes or supercomplexes *via* direct or indirect physical interactions, or membrane domains with special lipid compositions and cytoskeletal support resistant to detergent disruption; and (3) physical colocalization *in situ* within a very short distance, which can be probed either directly with super resolution (under 100 nm) microscopy or more conveniently and indirectly *via* a nanometer distance-sensitive imaging analysis method such as förster resonance energy transfer (FRET) or proximity ligation assay (PLA) (see section “BK-NMDAR Colocalization and Functional Coupling Within the Nanodomain”). We review the evidence and findings from these 3 directions in support of nanodomain coupling of BK channels with a specific Ca^2+^ channel.

## BK-Ca_V_ Coupling

### Ca_V_ Channels

Of the different types of Ca^2+^ channels, voltage-gated calcium (Ca_V_) channels play a vital role in providing intracellular Ca^2+^ ions to K_Ca_ channels. Ca_V_ channels are formed by central pore-forming α_1_ subunits and regulatory auxiliary subunits and are responsible for membrane depolarization–induced Ca^2+^ entry into excitable cells (Zamponi et al., 2015; Nanou and Catterall, 2018). The auxiliary subunits, which contribute to calcium channel diversity, are encoded by 4 α2δ genes, 4 β-subunit genes, and 9 γ-subunit genes. The α_1_ subunit forms the Ca^2+^ selective pore and consists of 24 TM α helices divided into 4 homologous domains, each containing 6 TM α helices (Wu et al., 2016) (Figure 1). Based on the electrophysiological and pharmacological properties of their ionic currents, Ca_V_ channels are classified into L-, N-, P/Q-, R-, and T-types. Based on the amino acid sequence similarities of the pore-forming α_1_ subunits, they are grouped into Ca_V_1, Ca_V_2, and Ca_V_3 types. L-type (Ca_V_1.1, Ca_V_1.2, Ca_V_1.3, and Ca_V_1.4) channels are activated by high voltages and distinguished by long-lasting (L) activation, i.e., slow voltage-dependent inactivation and blocked by calcium antagonist drugs such as dihydropyridines and phenylalkylamines. L-type channels have diverse functions, including the initiation of contraction in muscle cells, hormone secretion in endocrine cells, and local calcium signaling for gene transcription. P/Q- (Ca_V_2.1), N- (Ca_V_2.2), and R- (Ca_V_2.3) type channels are also activated by high voltages but have faster voltage-dependent inactivation. They are found primarily in neurons and are blocked by peptide toxins from spiders and snails. T-type (Ca_V_3.1, Ca_V_3.2, and Ca_V_3.3) channels are activated by low voltages (negative membrane potentials) and have fast deactivation upon repolarization and fast voltage-dependent inactivation. They are predominantly found in cardiac myocytes, sinoatrial nodes, and thalamic neurons.

### BK-Ca_V_ Functional Coupling

Researchers studying BK channels from different types of neurons have found that channel activity depends on Ca^2+^-influx through Ca_V_ channels and that blocking of the L-type (Storm, 1987; Prakriya and Lingle, 1999; Vivas et al., 2017), P/Q type (Edgerton and Reinhart, 2003; Womack et al., 2004), and N-type Ca_V_ channels (Marrion and Tavalin, 1998; Loane et al., 2007) with subunit-specific toxins suppresses neuronal BK channel currents. In addition, they discovered that BK-Ca_V_ coupling must occur within a nanodomain because the Ca_V_-dependent BK channel activation was rapid (e.g., ∼1 ms) and not disrupted by the Ca^2+^ chelator EGTA when patch-clamp recordings were performed on channels heterologously expressed in *Xenopus* oocytes, Chinese hamster ovaries (CHO), and tsA-201 cells (Berkefeld et al., 2006; Berkefeld and Fakler, 2008; Vivas et al., 2017), native channels in chromaffin cells (Prakriya and Lingle, 2000; Berkefeld et al., 2006), or neurons (Gola and Crest, 1993; Robitaille et al., 1993; Edgerton and Reinhart, 2003; Sun et al., 2003; Muller et al., 2007; Vivas et al., 2017). During functional coupling, the apparent voltage dependence, current kinetics, and amplitude of BK channel activation are affected by the voltage dependence, conductance, and coupling strength of the Ca_V_ channels. Thus, different Ca_V_ channels confer different apparent gating properties for BK channel activation (Berkefeld et al., 2006; Vivas et al., 2017). The lower-voltage–activated Ca_V_1.3 caused a much greater shift toward negative voltage in the activation of the BK channel than in that of the Ca_V_2.2 channel (Vivas et al., 2017). Based on EGTA’s and BAPTA’s effects on the Ca_V_2.1-induced BK channel’s current amplitude and time course recorded in the heterologous expression system, the distance between these 2 channels was estimated to be approximately 10–15 nm (Berkefeld et al., 2006). Similarly, the diffusional distance for Ca^2+^ ions from the Ca_V_ channels to the BK channels in hippocampal granule cells was estimated to be 13 nm per linear approximation of buffer Ca^2+^ diffusion (Muller et al., 2007). The T-type Ca_V_3.2 channel also provided Ca^2+^ for BK channel activation in a heterologous expression system of tsA-201 cells and in rat medial vestibular neurons (Rehak et al., 2013). However, the Ca_V_3-evoked BK channel currents were sensitive to EGTA, suggesting weaker microdomain coupling than that observed for nanodomain coupling with Ca_V_1.2, 1.3, 2.1, and 2.2 channels.

### Molecular Organization of BK-Ca_V_ Nanodomain Coupling

Affinity purification of BK channels in rat brains with anti-BKα antibodies and subsequent mass spectrometry analyses identified the formation of macromolecular protein complexes between BK channels and Ca_V_1.2 (L-type), Ca_V_2.1 (P/Q-type), and Ca_V_2.2 (N-type) channels (Berkefeld et al., 2006). Of the Ca_V_ channel proteins, Ca_V_2.1 was the most abundantly copurified with BKα. The auxiliary subunits of BKβ2, BKβ4, Ca_V_β1b, Ca_V_β2, Ca_V_β3, and Ca_V_β4 were also identified in the complexes. By coimmunoprecipitation, BKα was found to form complexes with Ca_V_1.2 and Ca_V_2.1 as well in the heterologous expression system of *Xenopus* oocytes (Berkefeld et al., 2006). The coimmunoprecipitation of BK channels and Ca_V_1.3 was also reported for native channels from rat brains (Grunnet and Kaufmann, 2004) and for heterologously expressed channels in tsA-201 cells (Vivas et al., 2017). These studies showed that BK and Ca_V_ channels formed complexes in both brain and heterologous expression systems, which rules out the requirement for neuron-specific protein to be present in order for the physical association to occur. Interestingly, the S0 TM segment of the BK channels was necessary for coimmunoprecipitation with Ca_V_3.2 in spite of the likely microdomain coupling of these two channels (Rehak et al., 2013).

Given the predicted close distance (10–15 nm) in functional coupling (Berkefeld et al., 2006; Muller et al., 2007) and the large sizes of BK and Ca_V_ channels, which are ∼13 and ∼10 nm wide (parallel to the membrane), respectively (Wu et al., 2015; Tao and MacKinnon, 2019) (Figure 1), they could be positioned in close contact *via* direct physical interactions. However, no protein domains, regions, or residues are known to be involved in BK-Ca_V_ interactions. Some reports support the possibility of indirect interactions between the BK and Ca_V_ channels. The colocalization of Ca_V_ and BK channels at the active zone of presynaptic nerve terminals was proposed to be mediated by the scaffold proteins, RIMs and RIMs binding proteins, which interact with Ca_V_ and BK channels, respectively (Sclip et al., 2018). Co-expression of the channels with a G protein-coupled receptor, β protein-co receptor, was reported to be needed for coimmunoprecipitation of BK Ca_V_1.2 channels in HEK293 cells (Liu et al., 2004), which is in contrast with the reported BK-Ca_V_1.2 complex formation without expression of any other exogenous protein in another heterologous system of *Xenopus* oocytes (Berkefeld et al., 2006). It is also unclear whether BK and Ca_V_ channels can directly interact with each other and form complexes in 1:1 stoichiometric relationships, as has been previously speculated (Berkefeld et al., 2006; Fakler and Adelman, 2008). Computational modeling of BK-Ca_V_ coupling at 1:1 (Cox, 2014) and other stoichiometric relationships (Montefusco et al., 2017) could reproduce the coupled electric activity observed in cells. A super-resolution microscopic study of BK and Ca_V_1.3 in a heterologous system and in rat hippocampal and sympathetic neurons revealed the formation of homotypic multichannel clusters of both Ca_V_1.3 and BK channels and a skewed, concentrated distribution of Ca_V_1.3 clusters occupying areas adjacent to BK clusters (Vivas et al., 2017). The BK channel clusters had a median area of 1,600 nm^2^ in tsA-201 cells, 2,000 nm^2^ in hippocampal neurons, and 2,800 nm^2^ in superior cervical ganglion neurons, containing roughly an average of 10–15 BK channels in a cluster. Most of the BK channel clusters were surrounded (within a radius of 200 nm) by a variable number (mean, ∼4) of Ca_V_1.3 clusters (median area, 1,600 nm^2^). Thus, surprisingly, the association between BK and Ca_V_1.3 channels occurred mainly in multichannel clusters and was not fixed in stoichiometric or geometric relationships, which was contradictory to the concept of 1:1 stoichiometry in physical interactions. In hippocampal and superior cervical ganglion neurons, small portions (3 and 10%, respectively) of the BK channel clusters contained Ca_V_1.3 channels, indicating the presence of some intimate BK-Ca_V_ coupling and interactions within a cluster. For BK-Ca_V_1.3 coupling *via* the observed heterogeneous, multichannel clustering, the local Ca^2+^ source for BK channel activation varies greatly according to the number and locations of the surrounding activating Ca_V_ channels. Interestingly, clustering facilitates the cooperative opening of the Ca_V_1.3 channels within a given cluster (Moreno et al., 2016), which could enhance BK-Ca_V_ functional coupling.

### Localization and Roles of BK-Ca_V_ Coupling in the Nervous System

**FIGURE 5:**
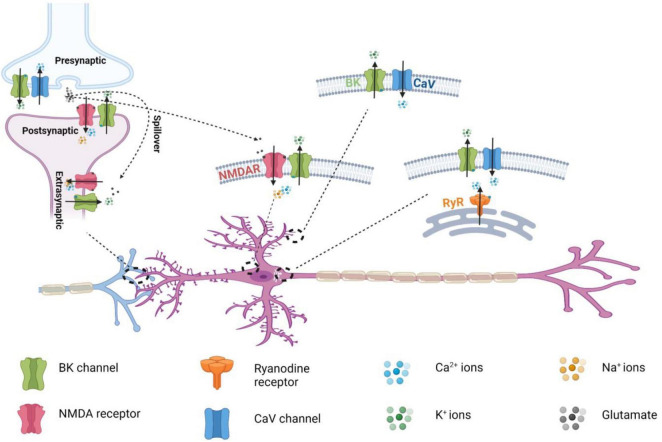
Distribution of the nanodomain coupling of BK channels with different Ca^2+^ permeable channels in neurons.

BK-Ca_V_ coupling has been recorded in a variety of different neurons and plays fundamental roles in the regulation of neural firing and transmission. The exceptionally large, single-channel K^+^ currents and tight BK-Ca_V_ coupling allow BK channels to function as potent negative-feedback regulators of both membrane potentials and Ca^2+^-influx. During the membrane depolarization–induced sequential activation of Ca_V_ and BK channels, BK channel-mediated large K^+^ currents rapidly counterbalance membrane depolarization and Ca^2+^ influx by repolarizing the membrane potential, which also limits further Ca^2+^ influx *via* the repolarization-induced closure of Ca_V_ channels. For BK-Ca_V_ nanodomain coupling, the voltage threshold of BK channel activation tends to reflect the voltage threshold of the coupled Ca_V_ channels (Vivas et al., 2017). If BK channels are activated by Ca_V_ channels before the membrane potential reaches the action potential firing threshold, BK-Ca_V_ coupling can prevent neuronal firing, as that implicated for BK-Ca_V_1.3 coupling in sympathetic neurons (Vivas et al., 2014, 2017). In most studies, the BK-Ca_V_ coupling was recorded on somatic membranes (Figure 5) and regulated neuronal firing by repolarizing action potentials and generating fAHP, such as those observed in Helix neurons (Gola and Crest, 1993), hippocampal neurons (Marrion and Tavalin, 1998), guinea pig sympathetic neurons (Davies et al., 1999), cerebellum Purkinje neurons (Edgerton and Reinhart, 2003), neocortical pyramidal neurons (Sun et al., 2003), hippocampal granule cells (Sun et al., 2003), and striatal cholinergic interneurons (Goldberg and Wilson, 2005). BK and Ca_V_ channels were also expressed on presynaptic terminals on which neurotransmitter release is tightly controlled by Ca^2+^ entry *via* Ca_V_ channels. The BK-Ca_V_ coupling at presynaptic terminals (Figure 5) negatively modulated the neurotransmitter release by negative feedback regulation of Ca_V_-mediated Ca^2+^ influx. Such a role for BK-Ca_V_ coupling as a controller of neurotransmitter release at presynaptic terminals has been demonstrated in frog neuromuscular junctions (Robitaille et al., 1993), Xenopus nerve–muscle synapses (Yazejian et al., 1997), and mouse motor nerve terminals (Protti and Uchitel, 1997). For the Ca_V_2.3 channel, it was previously reported to be absent in the isolated rat brain BK-Ca_V_ complexes and also failed in activating BK channels in the presence of EGTA in the heterologous expression system (Berkefeld et al., 2006). A later study showed co-immunoprecipitation of BK channels and Ca_V_2.3 from mouse hippocampus and deletion of Ca_V_2.3 in CA1 pyramidal cells reduced the BK channel function resulting in altered action potential waveforms which strengthens the synaptic transmission between CA1 and subiculum and increased the short-term plasticity (Gutzmann et al., 2019).

## BK-NMDAR Coupling

### NMDARs

NMDARs are ligand- and voltage-gated, Ca^2+^-permeable cation channels that function as coincidence detectors of elevated extracellular glutamate levels and membrane depolarization. Channel opening in NMDARs requires coincident glutamate binding–evoked channel activation and the membrane depolarization–induced release of pore blockades caused by extracellular Mg^2+^. NMDAR activation results in prolonged Na^+^ and Ca^2+^ influx that is critical to excitatory synaptic transmission and synaptic plasticity. Unlike the two other classes of ionotropic glutamate receptors, AMPA receptors (AMPARs) and kainate receptors (KARs), NMDARs are characterized by high Ca^2+^ permeability, slow deactivation, high affinity for glutamate, high unitary conductance (30–50 pS), and voltage-dependent blockading by extracellular Mg^2+^ (Zito and Scheuss, 2009; Paoletti, 2011; Paoletti et al., 2013). NMDARs are heterotetrameric channels composed of two obligatory GluN1 subunits and 2 modulatory GluN2 (A-D) or GluN3 (A–B) subunits (Figure 1). Each NMDAR subunit consists of 4 structural domains: 2 extracellular, tandem, globular, clamshell-like domains [the amino-terminal domain (ATD) for subunit assembly and the ligand-binding domain (LBD)] for the binding of glycine and glutamate; an ion channel pore TM domain made of 3 TM segments; and a highly variable, intracellular carboxyl-terminal domain (CTD) (Karakas and Furukawa, 2014) (Figure 1). NMDARs are involved in many aspects of synaptic transmission, dendritic integration, synaptic and neuronal maturation, and synaptic plasticity throughout the brain (Zito and Scheuss, 2009). They are also involved in various neurological and psychiatric diseases, including NMDAR hyperactivity–induced acute excitotoxicity (e.g., epilepsy and ischemic stroke) and chronic neurodegeneration (e.g., Alzheimer, Parkinson, and Huntington diseases and amyotrophic lateral sclerosis), NMDAR hypofunction–related neurodevelopmental disorders (e.g., schizophrenia), and many others (e.g., pain, depression, autism, white matter injury, and anti-NMDAR encephalitis) (Lakhan et al., 2013; Paoletti et al., 2013; Zhou and Sheng, 2013; Bourinet et al., 2014). In spite of their ubiquitous expression in the mammalian central and peripheral nervous systems, BK channels and NMDARs were traditionally considered to be localized in different compartments as presynaptic and postsynaptic channels, respectively. However, in an early 2001 study, BK channels were activated by NMDAR-mediated Ca^2+^ influx at extrasynaptic sites in rat olfactory bulb granule cells; this was a previously unknown neuronal activity-inhibitory role for NMDARs (Isaacson and Murphy, 2001).

Compared to the intensive research on BK-Ca_V_ coupling, the attention to BK-NMDAR coupling had been lacking. Questions regarding whether BK channels and NMDARs can directly associate to form protein complexes, whether such extrasynaptic BK-NMDAR coupling is also present in other neurons or brain regions, and whether this coupling occurs in other subcellular location were not addressed until more recently.

### Biochemical Basis of BK-NMDAR Interactions

Affinity purification and mass spectrometry analyses of immunopurified BK channels and NMDA receptors from rat brains have found that they were mutually identified together (Zhang et al., 2018). Tandem immunopurification with anti-BKα and ant-GluN1 antibodies in the first and second rounds of purification, respectively, was able to purify the protein complexes containing both BKα and GluN1. The copurification of GluN2A and GluN2B with the BKα-GluN1 complex indicates the association of BK channels with functional NMDARs. In the heterologous expression system of HEK293 cells, it was found that BKα specifically interacts with the NMDARs’ obligatory GluN1 subunit, whereas there were no interactions observed between BKα and the NMDARs’ GluN2A and GluN2B, the kainate receptors’ GluK1 and GluK2, or the AMPA receptors’ GluR1 and GluR2. With truncation constructs of BKα and GluN1 and chimeric constructs of GluN1/GluK2, the BKα-GluN1 interactions involved the former’s S0–S1 long loop and the latter’s TM regions, including the loops and cytosolic C-terminus. An engineered protein containing the GluN1’s cytosolic regions (residues 563–587 for the M1–M2 loop region and residues 813–920 for the C-terminus), which was expressed in and purified from *Escherichia coli*, was able to interact directly with a synthesized peptide of the BKα S0–S1 loop region (residues 46–93) *in vitro*. Furthermore, the synthesized peptide of the S0–S1 loop region competitively disrupted the association between BKα and GluN1. These findings indicate that BKα and GluN1 directly interact with each other *via* their cytosolic regions, including the BKα S0–S1 loop region (Figure 1), which seems to be flexible in structure as its structure was undefined in the reported cryo-EM structures of the BK channels (Tao and MacKinnon, 2019).

### BK-NMDAR Colocalization and Functional Coupling Within the Nanodomain

The colocalization of BK and NMDARs was probed with an *in situ* PLA (Zhang et al., 2018; Gomez et al., 2021). PLA displays point-like staining signals only when the 2 epitopes on the interacting proteins for primary antibodies are in proximity (<40 nm) (Soderberg et al., 2006), making PLA a suitable assay for the detection of protein colocalization within the nanodomain. With *in situ* PLA, BKα and GluN1 were found to be colocalized in both the heterologous expression system and, broadly, in different regions of mouse hippocampus (Zhang et al., 2018). Consistent with the sole requirement of GluN1 for protein complex formation with BK channels (Zhang et al., 2018), PLA with HEK293 cells co-expressing BK channels with GluN1/GluN2A or GluN1/GluN2B showed no preference in BK channels’ proximity to these 2 types of NMDARs (Gomez et al., 2021). In the dentate gyrus, PLA signals of the BKα-GluN1 complexes were most abundant in the molecular layer region (Zhang et al., 2018), which is consistent with the predominant dendritic distribution of NMDARs. Glutamate-induced BK channel outward currents were observed when whole-cell voltage clamp recording was performed on mature dentate gyrus granule cells in mouse hippocampal slices upon application of glutamate toward soma (Zhang et al., 2018). The currents were blocked by the NMDAR antagonist (2R)-amino-5-phosphonovaleric acid (AP5) and pore-blocker MK-801, indicating involvement of the receptor’s ion-conducting function. The glutamate-induced BK channel currents were insensitive to intracellularly applied EGTA but became significantly smaller in the presence of BAPTA, confirming nanodomain functional coupling of BK channels with NMDARs. Intracellular application of the synthesized S0–S1 loop peptide reduced the glutamate-induced BK channel outward currents, indicating that the physical interactions between these 2 channels play a role in their functional coupling. EGTA-insensitive coupling of BK channels with NMDARs was also recently observed in a subset of barrel cortex layer 5 pyramidal neurons (BC-L5PNs) but when NMDA was applied toward basal dendrites instead of soma (Gomez et al., 2021). In a heterologous expression system of HEK293 cells, similar results of BK-NMDAR (GluN1/GluN2A) functional coupling were revealed by whole cell voltage-clamp recording (Zhang et al., 2018). In excised inside-out membrane patches of HEK293 cells, BK channel openings at the single channel level were observed upon NMDAR activation using flash photolysis of caged NMDA (Zhang et al., 2018). In HEK293 cells, activation of NMDARs formed by GluN1/GluN2A or GluN1/GluN2B both resulted in large shifts in the apparent voltage dependence of BK channel activation (Gomez et al., 2021).

### Localization and Roles of BK-NMDAR Coupling in the Nervous System

Both BK channels and NMDARs are widely expressed in different regions of the central and peripheral nervous system. Using a pull-down assay, Zhang et al. (2018) showed that BK-NMDAR complexes are ubiquitously present in the brain, as BKα and GluN1 mutually pulled down each other in all examined brain regions, including the hippocampus, cerebellum, cortex, thalamus, striatum, and olfactory bulb. The subcellular distribution of NMDARs is affected by their subunit composition. In the adult central nervous system, GluN2B is mainly expressed at extrasynaptic sites, whereas GluN2A-containting receptors are enriched at postsynaptic sites at synapses (Paoletti et al., 2013). Given that both GluN2A and GluN2B are present in neuronal BK-NMDAR complexes, these complexes are expected to exist both extrasynaptically and postsynaptically. NMDAR-induced BK channel activation has been observed at the extrasynaptic sites of olfactory bulb granule cells (Isaacson and Murphy, 2001) and dentate gyrus granule cells (Zhang et al., 2018). At extrasynaptic sites, functional BK-NMDAR coupling can be induced by accumulated glutamate spillover during repetitive synaptic activities (Figure 4), such as those observed in olfactory bulb granule cells (Isaacson and Murphy, 2001). At postsynaptic sites, NMDAR-coupled BK channels can be immediately activated by presynaptically released glutamate during synaptic transmission (Figure 5). BK channels are generally considered to be axonal channels (Misonou et al., 2006), but a significant portion has also been observed at postsynaptic terminals in the stratum radiatum and oriens of the rat hippocampus (Sailer et al., 2006). However, the mechanism of channel activation and the function of BK channels at postsynaptic sites were largely unknown until recently. Interestingly, with BKα knockout mice and pharmacological tools, BK channels were found to negatively regulate the excitatory postsynaptic potentials (EPSPs) of dentate gyrus granule cells evoked by single-pulse stimulation of presynaptic fibers from the in the middle of the molecular layer (Zhang et al., 2018). Such a regulatory effect of BK channels on synaptic transmission at perforant path–dentate granule cell synapses was found to be insensitive to EGTA and strictly dependent on NMDAR activity. Given that the BK-NMDAR coupling was induced by a single pulse stimulation, the coupling must occur within the immediate reach of newly released glutamate in the synaptic cleft, i.e., at the pre- or postsynaptic terminal. A presynaptic effect of the BK channels was largely ruled out because of the lack of effect of paxilline and BKα knockout on presynaptic transmitter release. The postsynaptic dendritic location of the BK-NMDAR coupling was confirmed by the blockade effects of postsynaptically loaded paxilline and MK-801. When loaded intracellularly, the synthetic S0–S1 peptide also abolished the BK channels’ effect on synaptic transmission, suggesting that physical interactions are necessary for functional coupling at postsynaptic terminals (Zhang et al., 2018). The postsynaptic BK-NMDAR interactions and coupling (Figure 5) provide a novel mechanism for the negative feedback regulation of synaptic transmission *via* NMDAR-mediated activation of postsynaptic BK channels by the presynaptically released neurotransmitter glutamate in the synaptic cleft. Dentate gyrus granule cells are the first checkpoints for cortical information entering the hippocampus, where learning and memory take place. The findings on NMDAR-mediated BK channel activation and the modulatory effect of postsynaptic BK-NMDAR coupling on synaptic transmission provide a new molecular basis for understanding the role of BK channels in hippocampal learning and memory (Matthews and Disterhoft, 2009; Ye et al., 2010; Typlt et al., 2013; Springer et al., 2014).

Postsynaptic BK-NMDAR coupling was also recently reported to occur in the basal dendrites of approximately 40% of BC-L5PNs (Gomez et al., 2021). The application of NMDA toward basal dendrites led to large NMDAR inward and BK channel outward currents recorded in soma, supporting dendritic locations of BK-NMDAR coupling (Gomez et al., 2021). Such large inward currents of NMDARs and outward currents of BK channels were typically observed when BK-NMDAR coupling occurred locally at the soma (Isaacson and Murphy, 2001; Zhang et al., 2018). Similar to that observed in dentate gyrus granule cells (Zhang et al., 2018), the NMDAR-mediated BK channel activity in the basal dendrites reduced the postsynaptic membrane potentials (Gomez et al., 2021). Interestingly, as compared to the BC-L5PNs lacking BK-NMDAR coupling, the basal dendrites displaying NMDAR-mediated BK activation had no Ca^2+^ spike and had shortened durations and reduced amplitudes of after hyperpolarization in current injection–elicited action potentials. Furthermore, these BC-L5PNs also had reductions in spike timing–dependent LTP (t-LTP). t-LTP levels were restored by blockading the BK channel with paxilline, and reductions in these levels were prevented when the frequency and number of pre- and postsynaptic stimulation pairings were increased (Gomez et al., 2021). It was proposed that the BK-NMDAR coupling increases threshold for induction of t-LTP by functioning as high-pass filters for incoming synaptic input (Gomez et al., 2021). These putative roles for postsynaptic BK-NMDAR coupling on the regulation of action potential and LTP are novel. However, it remains to be determined whether NMDARs and their nanodomain coupling to BK channels are the major contributors to the BK channel activities underlying the observed differences between these 2 types of BC-L5PNs. Application of the synthetic S0–S1 peptide to decouple BK-NMDAR interactions (Zhang et al., 2018) could potentially answer this question.

The recent discovery of BK-NMDAR complex formation and functional coupling helps explain previous reports of NMDAR-mediated, apamin-insensitive K_Ca_ currents in cultured, postnatal rat hippocampal neurons (Zorumski et al., 1989); the need for both NMDAR and BK channel activities in the inhibition of opioid release in the spinal dorsal horn (Song and Marvizon, 2005); and BK channel–mediated negative feedback on NMDAR-mediated dendritic spine Ca^2+^ transients in the cartwheel cells of the dorsal cochlear nucleus (He et al., 2014).

### Comparison of BK-Ca_V_ and BK-NMDAR Coupling

Compared with BK-Ca_V_ coupling, BK-NMDAR coupling has distinct kinetic and functional properties. It produces unique neurotransmitter release–dependent K^+^ signaling. NMDARs provide more sustained Ca^2+^ sources for BK channel activation than do Ca_V_ channels because of the slow rate of NMDAR deactivation (τ = ∼40 ms–2 s). Functional coupling of BK channels with NMDARs at extrasynaptic sites results in glutamate spillover–induced, extrasynaptic BK channel activation and neuronal activity inhibition. Postsynaptic NMDARs coincidently detect presynaptic glutamate release and postsynaptic membrane depolarization and can thus facilitate BK channel activation in a spike timing–dependent manner owing to the intrinsic properties of NMDAR-mediated Ca^2+^ influx. The resultant BK channel K^+^ currents reduce the amplitude of EPSPs and promote Mg^2+^ blockade of NMDARs by repolarizing postsynaptic membranes. Given the involvement of BK channels in learning and memory (Matthews and Disterhoft, 2009; Ye et al., 2010; Typlt et al., 2013; Springer et al., 2014), BK channels may regulate long-term potentiation or depression *via* postsynaptic BK-NMDAR coupling. It is of note that the other types of K_Ca_ channels, e.g., SK channels, are also reported to be functionally coupled with Ca_V_ channels and NMDA receptors (NMDARs) (Marrion and Tavalin, 1998; Ngo-Anh et al., 2005; Wang et al., 2014). However, there is scarce evidence on the physical complex formation between Ca^2+^ channels and SK or IK channels, which is consistent with the lack of the requirement for strict, close colocalization for functional coupling. In spite of the physiological significance, the coupling of IK and SK channels with Ca^2+^-permeable channels is beyond the focus of this review. Previous reviews of this topic can be found elsewhere (Fakler and Adelman, 2008; Gueguinou et al., 2014).

## Coupling of BK Channels With Other Channels

### Coupling of BK Channels With Intracellular Ca^2+^-Release Channels

Ca^2+^-release channels are intracellular Ca^2+^ channels responsible for the release of Ca^2+^ from endoplasmic and sarcoplasmic reticulum (ER and SR) which form the intracellular Ca^2+^ stores (Woll and Van Petegem, 2022). They are giant membrane proteins consisting of two evolutionarily related gene families, ryanodine receptors (RyRs) and inositol-1,4,5-trisphosphate receptors (IP_3_Rs). The three RyR isoforms (RyR1, RyR2, and RyR3) are each ∼ 5,000 amino acid residue polypeptides that assemble into ∼2.2 MDa homotetrameric channels. They are expressed in different tissues including brains. As they are mostly studied in the context of muscle contraction, RyR1 is known as the skeletal muscle isoform and RyR2 as the cardiac isoform. RyR3 is ubiquitously expressed. The three IP_3_R isoforms (IP_3_R1, IP_3_R2, and IP_3_R3) are each ∼2,700 amino acid residue polypeptides that assemble into ∼1.2 MDa homotetrameric or heterotetrameric channels. IP_3_Rs have a broad tissue distribution with a high abundance in the cerebellum. Both RyRs and IP3Rs are sensitive to changes in cytosolic Ca^2+^ concentrations in that the channels are stimulated by rises in the cytosolic Ca^2+^ concentration but inhibited by high cytosolic Ca^2+^ concentration. RyRs are activated by Ca^2+^-influx mediated by plasma membrane Ca_V_ channels *via* a mechanism of Ca^2+^-induced Ca^2+^ release (CICR). IP3Rs are activated by the cytosolic IP3 molecule formed by protein lipase C (PLC) through hydrolysis of phosphatidylinositol 4,5-bisphosphate (PIP2) into IP_3_ and diacylglycerol (DAG). PLC is activated through a G-protein-coupled receptor (GPCR) or receptor tyrosine kinase (RTK) signaling pathway.

BK and Ca_V_ channels on somatic plasma membranes (PM) couple to RyRs on ER to form double (Ca_V_-RyR and RyR-BK) PM-ER nanodomains in Ca^2+^ signaling in the cartwheel inhibitory interneurons of the dorsal cochlear nucleus (Irie and Trussell, 2017). The triad Ca_V_/RyR/BK channel coupling (Figure 5) was reported to be EGTA-resistant and rapid (within the time of a single spike), and thus provided a mechanism for the rapid control of action potentials on a millisecond timescale (Irie and Trussell, 2017). Immunofluorescence analysis showed partial overlap of puncta of BK and RyR labeling in mouse cartwheel cells (Irie and Trussell, 2017). In mouse dentate gyrus granule neurons, knockout of the BK channel β4 subunit caused increased functional coupling between RyR and BK channels, resulted in an increase in the fAHP amplitude (Wang et al., 2016). A similar effect was observed by knockin of the seizure-prone gain-of-function (R2474S) RyR mutant channels. This study revealed different roles of the BK-Ca_V_ and BK-RyR coupling during action potential in that BK channel activation is dependent on L-type Ca_V_ channels in repolarization phase but RyRs during fAHP (Wang et al., 2016). In cerebral smooth muscle cells, another type of triad channel coupling, TRPV4/RyR/BK, has been reported for RyR-induced BK channel activation (Earley et al., 2005). Activation of TRPV4 by 11,12 EET in freshly isolated cerebral myocytes led to elevated Ca^2+^ spark from RyRs and transient BK activity which was unaffected by inhibition of Ca_V_ channels. The TRPV4-RyR-BK channel coupling accounts for smooth muscle hyperpolarization and arterial dilation *via* Ca^2+^-induced Ca^2+^ release in response to TRPV4 activation by an endothelial-derived factor (Earley et al., 2005). However, whether the coupling also occurs within nanodomains is unclear, as no Ca^2+^ chelators were used to probe the strengths of the Ca^2+^ coupling.

Potential functional coupling of BK channels with IP_3_Rs was reported in glioma cells *via* colocalization within lipid rafts (Weaver et al., 2007). With whole cell patch-clamp recording, it was observed that the voltage-induced BK channel activity was reduced upon lipid raft disruption with methyl-β-cyclodextrin. Pretreatment of glioma cells with thapsigargin to deplete the intracellular Ca^2+^ store or 2-aminoethoxydiphenyl borate to inhibit IP3Rs negated the effect of methyl-β-cyclodextrin. Stimulation of muscarinic acetylcholine receptors (mAChRs) with muscarine or acetylcholine (ACh), which was known to promote IP3 formation, elicited an increase in [Ca^2+^]_i_ that subsequently activated BK channels and caused cell hyperpolarization. Disruption of lipid rafts or inhibition of IP3Rs prevented the ACh-induced rise in [Ca^2+^]_i_ and the BK channel-induced hyperpolarization of the membrane. Both BK channels and IP_3_Rs were found to associate or localize with lipid rafts as detected by immunoblot in the isolated lipid raft fractions or by immunofluorescence on cells. Given the presence of 10 mM EGTA in the pipette solution of the whole cell recording and the sensitivity to Ca^2+^ store depletion, it is likely that the BK-IP_3_R coupling involves Ca^2+^ and the coupling exists within nanodomain. However, the lack of data for comparison with/without EGTA or BAPTA prevents drawing a solid conclusion. Another instance of potential BK-IP_3_Rs interactions was observed in rat and mouse cerebral artery smooth muscle cells (Zhao et al., 2010). Activation of BK channels by IP_3_ or its membrane-permeable analog occurred in both intact cells and excised membrane patches *via* cell-attached and inside-out patch-clamp recording configurations, respectively. Inhibition of IP_3_Rs, knockout of IP_3_R1, or application of an IP_3_R1 antibody suppressed the IP_3_-induced BK channel activation in inside-out recording of excised membrane patches. Immuno-FRET imaging analysis indicated BK-RyR1 colocalization. The BK channel α and β1 subunit were detected to be coimmunoprecipitated with RyR1. However, the IP_3_-induced BK channel activation in inside-out recording was time-independent after membrane patch excision and was insensitive to both BAPTA and EGTA, indicating some SR Ca^2+^-release independent mechanism. In another conflicting report, the IP3 was found to activate BK channel in pig coronary artery smooth muscle cells *via* an IP_3_R-indepenent mechanism (Yang et al., 2013). Therefore, uncertainty exists whether there is Ca^2+^-mediated BK-IP_3_R coupling and caution is needed in interpretation of the data on IP_3_-related BK channel activation.

### Coupling of BK Channels With Transient Receptor Potential Channels

BK channels have also been reported to functionally couple with TRP channels. TRP channels are a superfamily of cation channels whose activation mechanisms are more diverse than those of any other group of ion channels. TRP channels play critical roles in sensory physiology, including vision, taste, olfaction, hearing, touch, and thermo- and osmosensation. The TRP superfamily is divided into 7 subfamilies: 5 group 1 TRPs (TRPC, TRPV, TRPM, TRPN, and TRPA) and 2 group 2 subfamilies (TRPP and TRPML). The transient receptor potential vanilloid receptor 1(TRPV1) channel is a non-selective cation channel activated by a variety of exogenous and endogenous physical and chemical stimuli, such as temperature and capsaicin. In the heterologous expression system of HEK293 cells, BK channels were found to be activated by Ca^2+^ influx through TRPV1 channels in a largely EGTA-insensitive manner (Wu et al., 2013). The TRPV1-induced BK currents were also observed in dorsal root ganglion (DRG) cells (Wu et al., 2013). Coimmunoprecipitation showed formation of the BK-TRPV1 complex in both HEK293 and DRG cells. There have also been reports of the functional coupling of BK channels with TRPV4 in human bronchial epithelial cell lines (Fernandez-Fernandez et al., 2008) and with TRPC1 in vascular smooth muscle cells (Kwan et al., 2009). Activation of TRPV4 by activator, osmotic and mechanical stimulation led to BK channel activities, which was lost upon of TRPV4 knockdown (Fernandez-Fernandez et al., 2008). Coimmunoprecipitation and immunofluorescence showed complex formation between BK and TRPC1 channels in HEK293 cells and their colocalization in vascular smooth muscle cells (Kwan et al., 2009). The BK-TRPC1 coupling was found to play a role in agonist-induced membrane depolarization and vascular contraction in isolated rat mesenteric arteries (Kwan et al., 2009). Whether the BK-TRPV4 and BK-TRPC1 couplings occurred within nanodomain is unknown, as no data on EGTA-sensitivity were presented in both studies (Fernandez-Fernandez et al., 2008; Kwan et al., 2009) and no protein complex formation and colocalization was demonstrated in the BK-TRPV4 coupling case (Kwan et al., 2009). Coimmunoprecipitation of BK with TRPC3 and TRPC6 channels was observed in differentiated podocyte cell line and heterologous expression system of HEK293 cells (Kim et al., 2009). TRPC3 but not TRPC6 was found to increase the surface expression of BKα subunit splice variant (Slo1_VEDEC_) (Kim et al., 2009). However, the possibility of Ca^2+^-mediated functional coupling of the BK with TRPC3 or TRPC6 channels was not addressed in this report (Kim et al., 2009).

## Discussion

Reported studies have clearly demonstrated the nanodomain functional couplings of BK channels with Ca^2+^-permeable channels, particularly the Ca_V_ and NMDAR channels, in a variety of different cells. These mechanisms of nanodomain couplings allow BK channels to play diverse cellular and physiological roles. Given the BK channel’s widespread expression and critical physiological roles, directly targeting it can have unavoidable adverse side effects. Selective enhancement or disruption of the interactions between BK channels and Ca^2+^ permeable channels using small chemicals, peptidomimetic molecules, or genetic methods can be an effective way to modify BK channel activity or to indirectly intervene in Ca_V_ or NMDAR function by limiting or enhancing the negative feedback from BK channels. It will be important to know how they organize and interact on membrane within nanodomains for effective functional coupling. However, the biochemical bases of the interactions of BK channels with Ca^2+^ permeable channels, either direct or indirect, remain mostly unknown. The recent advances in the cryo-EM–based structural determination of ion channels have given us a better understanding of the structure-function relationship of ion channels. Future determination of the structures of whole coupling complexes should enable researchers to develop potent and specific inhibitors or activators of these complexes and thus design novel therapeutic interventions. The initial investigation into the biochemical basis of BK-NMDAR coupling found direct physical interactions between the BK and NMDAR intracellular regions and domains (Zhang et al., 2018). Encouragingly, the synthetic S0–S1 peptide of BK channels was shown to be effective in disrupting the BK-NMDAR interactions and couplings (Zhang et al., 2018).

BK-Ca_V_ nanodomain coupling intertwines the functions of these two types of channels in the regulation of neuronal excitability. Compared to the cellular and physiological roles of BK-Ca_V_ coupling, those of BK-NMDAR coupling in different cells are less studied and understood. Postsynaptic and extrasynaptic BK-NMDAR couplings (Figure 5) provide unique neurotransmitter-dependent Ca^2+^ sources for BK channel activation and function, which could be important for synaptic plasticity, as was noted in the recent study (Gomez et al., 2021). More studies in different brain regions or neurons under normal or pathological conditions could expand our understanding of the physiological and pathological roles of BK-NMDAR coupling. NMDARs play numerous physiological and pathological roles. The extent to which BK-NMDAR coupling contributes to NMDAR-mediated Ca^2+^ signaling remains to be determined. The development of pharmacological tools that specifically interrupt BK-NMDAR interactions, such as the synthetic S0–S1 peptide (Zhang et al., 2018), will be helpful in determining the physiological and pathological roles of BK-NMDAR coupling. For the structural and functional coupling of BK channels with other Ca^2+^ permeable channels within nanodomain, the reported studies remain sparse or very limited in evidence. More studies will be needed to establish the structural and functional coupling, determine the underlying biochemical mechanisms, and understand the physiological roles.

## Author Contributions

All authors listed have made a substantial, direct, and intellectual contribution to the work, and approved it for publication.

## Conflict of Interest

The authors declare that the research was conducted in the absence of any commercial or financial relationships that could be construed as a potential conflict of interest.

## Publisher’s Note

All claims expressed in this article are solely those of the authors and do not necessarily represent those of their affiliated organizations, or those of the publisher, the editors and the reviewers. Any product that may be evaluated in this article, or claim that may be made by its manufacturer, is not guaranteed or endorsed by the publisher.
